# Using a monitoring and evaluation framework to improve study efficiency and quality during a prospective cohort study in infants receiving rotavirus vaccination in El Alto, Bolivia: the Infant Nutrition, Inflammation, and Diarrheal Illness (NIDI) study

**DOI:** 10.1186/s12889-017-4904-5

**Published:** 2017-11-28

**Authors:** Anna M. Aceituno, Kaitlyn K. Stanhope, Paulina A. Rebolledo, Rachel M. Burke, Rita Revollo, Volga Iñiguez, Parminder S. Suchdev, Juan S. Leon

**Affiliations:** 1 RTI International, 3040 E. Cornwallis Road, PO Box 12194, Research Triangle Park, NC 27709 USA; 2 Hubert Department of Global Health, Rollins School of Public Health, Emory University, Atlanta, GA USA; 3 Department of Epidemiology, Rollins School of Public Health, Emory University, Atlanta, GA USA; 4 Emory School of Medicine, Atlanta, GA USA; 5 Servicio Departamental de Salud, La Paz, Bolivia; 6 Instituto de Biotecnología y Microbiología, Universidad Mayor de San Andrés, La Paz, Bolivia; 7 Nutrition Branch, Centers for Disease Control and Prevention, Atlanta, GA USA

## Abstract

**Background:**

Implementing rigorous epidemiologic studies in low-resource settings involves challenges in participant recruitment and follow-up (e.g., mobile populations, distrust), biological sample collection (e.g., cold-chain, laboratory equipment scarcity) and data collection (e.g., literacy, staff training, and infrastructure). This article describes the use of a monitoring and evaluation (M&E) framework to improve study efficiency and quality during participant engagement, and biological sample and data collection in a longitudinal cohort study of Bolivian infants.

**Methods:**

The study occurred between 2013 and 2015 in El Alto, Bolivia, a high-altitude, urban, low-resource community. The study’s M&E framework included indicators for participant engagement (e.g., recruitment, retention, safety), biological sample (e.g., stool and blood), and data (e.g., anthropometry, questionnaires) collection and quality. Monitoring indicators were measured regularly throughout the study and used for course correction, communication, and staff retraining.

**Results:**

Participant engagement indicators suggested that enrollment objectives were met (461 infants), but 15% loss-to-follow-up resulted in only 364 infants completing the study. Over the course of the study, there were four study-related adverse events (minor swelling and bruising related to a blood draw) and five severe adverse events (infant deaths) not related to study participation. Biological sample indicators demonstrated two blood samples collected from 95% (333 of 350 required) infants and stool collected for 61% of reported infant diarrhea episodes. Anthropometry data quality indicators were extremely high (median SDs for weight-for-length, length-for-age and weight-for-age z-scores 1.01, 0.98, and 1.03, respectively), likely due to extensive training, standardization, and monitoring efforts.

**Conclusions:**

Conducting human subjects research studies in low-resource settings often presents unique logistical difficulties, and collecting high-quality data is often a challenge. Investing in comprehensive M&E is important to improve participant recruitment, retention and safety, and sample and data quality. The M&E framework from this study can be applied to other longitudinal studies.

**Electronic supplementary material:**

The online version of this article (10.1186/s12889-017-4904-5) contains supplementary material, which is available to authorized users.

## Background

The challenges in implementing epidemiologic studies in low-resource settings, particularly with infants, include effectively engaging with mobile communities [1], recruiting and training qualified staff [2], avoiding cold-chain or lab supply disruption [3], ensuring quality equipment for sample processing, minimizing burden to participants, and building relationships and communicating between multi-national study teams [4]. Though the methods of such studies are sometimes reported [5–7], strategies and systems for maintaining data quality and knowledge exchange with local partners are not well documented.

Monitoring and evaluation (M&E) frameworks offer a metric for tracking progress towards project goals through a logical framework documenting intermediate and long-term measurable objectives [8, 9]. Intermediate objectives are ways to check on individual pieces of a study and are measured once or more at pre-set intervals during implementation, allowing study staff to reassess implementation strategy if objectives are not met. Long-term objectives clarify plans for the study [8]. M&E frameworks facilitate adaptation to real-world conditions during study implementation, improving data quality, participant safety, and study efficiency. Participant engagement (e.g., recruitment, retention, safety), biological sample (e.g., stool and blood), and data (e.g., anthropometry, questionnaires) collection and quality during human subjects research with vulnerable populations or in low-resource settings.

Rotavirus (RV) is the most common cause of severe diarrhea in infants worldwide [10, 11], despite widespread implementation of anti-RV oral vaccines Rotarix® and Rotateq®. These vaccines are least efficacious and effective in developing countries where morbidity and mortality from RV are high [12–15]. Proposed hypotheses for reduced oral vaccine effectiveness include growth impairment, micronutrient deficiency in infants and mothers, and inflammation due to co-infection with other enteric pathogens [16–25]. Few studies have tested hypothesized causes for reduced RV vaccine effectiveness in low-resource settings [18]. Testing these hypotheses requires human subjects’ studies in low-resource settings, where the vaccines are least effective. The additional logistical challenges of conducting human subjects research in low-resource settings with vulnerable populations (infants) could interfere with data quality and participant safety. A robust M&E framework may be helpful to continuously monitor and improve participant engagement, biological sample, and data quality and collection.

This paper describes the use of a monitoring and evaluation (M&E) framework to improve study efficiency and quality during participant engagement, and biological sample and data collection in human subjects research in a low-resource setting. We use as a case study an observational prospective cohort study to evaluate the effect of infant chronic undernutrition on infant RV-specific immunogenicity of 364 infants in El Alto, Bolivia.

## Methods

### Study protocol

The primary objective of the Infant Nutrition, Immunology, and Diarrheal Illness study (Nutrición, Inmunología y Diarrea Infantil - NIDI study) was to evaluate the effect of infant chronic undernutrition (length-for-age z-score (LAZ) < −2 or weight-for-length z-score (WLZ) < −2 [26]) on infant RV-specific immunogenicity of infants in El Alto, Bolivia. Secondary objectives were to evaluate the effects of (1) maternal and infant micronutrient deficiency, (2) maternal RV-specific immunity, and (3) early-life enteric infection and inflammation on infant RV-specific immunogenicity. A tertiary objective was to evaluate the effects of early-life enteric infection, subclinical inflammation, nutritional status, and post-vaccination RV-specific immunity on malnutrition at one year of age.

Bolivia was selected because it has a high prevalence of infant and maternal anemia and undernutrition, high incidence of diarrheal illness, high under-five mortality rate [27], and because it provides, as part of state-sponsored programming, the Rotarix® vaccine free of charge during well-child visits. Following a pilot study in three hospitals, the city of El Alto was selected as a location with proximity to a partner laboratory and home to a marginalized indigenous population with chronic undernutrition.

A sample size of 350 sets of maternal-infant data was calculated to provide 80% power (2-sided α ≤ =0.05), based on 1) an 18% difference between rates of RV-vaccine antibody seroconversion among children with vs. without chronic undernutrition [12–14, 28], and 2) an estimated prevalence of 29–38% chronic undernutrition in mothers and infants. Unpublished pilot data collected 2010–2011 at the study hospitals found that 29% of children less than four months of age had moderate to severe chronic undernutrition undernutrition (LAZ < −2 or WLZ < −2 [26]) and the 2008 Bolivia National Demographic and Health Survey found that 38% of women had anemia (Hemoglobin (Hb) <14.7 g/dl) [27]. Based on an estimated 22% loss to follow-up (LTFU) or failed blood sample collection (unpublished pilot data), the recruitment objective was set at 450 mother-infant (M-I) pairs.

Data were collected June 2013 – March 2015. There were up to ten study visits; seven or eight scheduled hospital visits at well-child visits over 12–18 months, and one or two at home 4 and/or 7 days after the first dose of the RV vaccine (Table 1). Some infants returned to the hospital for an eighth visit for a third blood sample collection if it was not collected at the seventh well-child hospital visit at one year of age. The first two study visits were prior to the initial dose of the RV vaccine and served as baseline data. Please see the first study visit questionnaire provided as an Additional file 1 (NIDI_Study_Questionnaire_Visit_1.pdf) for complete baseline information collected.

**Table 1:** The NIDI study visit and data collection schedule 2012–2015

Measure	Study Visit (Infant Age in Months)							
Target visit schedule	1 (1)	2 (2)	3+ (2)	4 (3)	5 (4)	6 (6–8)	7 (9)	8+ (12–18)
RV^a^ vaccine criteria	–	–	4 and/or 7 days post RV dose 1	–	–	60 days post RV dose 2	–	–
Maternal Characteristics								
Weight and height	X	X		X	X	X	X	X
Clinical and prenatal history	X							X
Prenatal and postpartum micronutrient supplementation	X							X
Two-week morbidity	X	X	X	X	X	X	X	X
Plasma anti-RV IgG^b^	X							
Plasma ferritin, sTfR^c^, CRP^d^, AGP^e^	X					X		
Maternal/Infant SES								
Sociodemographic	X							X
Household characteristics	X							X
Infant feeding practices, iron supplementation	X	X	X	X	X	X	X	X
Infant Nutritional & Inflammation Status								
Anthropometry	X	X		X	X	X	X	X
Vitamin A, zinc, micronutrient sprinkles supplementation						X		X
Plasma ferritin, sTfR^b^, zinc, CRP^d^, AGP^e^		X				X		X
Infant Infection and Morbidity								
Two-week morbidity and diarrhea recall	X	X	X	X	X	X	X	X
Vaccines received						X		X
Fecal RV	X	X	X					
Fecal RV and enteric co-pathogens	When diarrhea reported							
Outcome: Infant RV Vaccine Immunogenicity								
Plasma anti-RV IgG		X				X		X
Plasma anti-RV IgA		X				X		X

^a^RV = Rotavirus

^b^IgG = Immunoglobulin

^c^sTfR = Soluble transferrin receptor

^d^ CRP = C-reactive protein

^e^AGP=α 1-acid glycoprotein

### Development of the monitoring and evaluation framework

The NIDI study M&E framework was developed using a formal M&E logical framework [29] based on the overall study goal and specific objectives and included a comprehensive data management plan and objectives in the areas of participant engagement (e.g., recruitment, retention, safety), biological sample (e.g., stool and blood), and data (e.g., anthropometry, questionnaires) collection and quality (Tables 2 and 3).

**Table 2:** Example Evaluation Indicators for Participant Enrollment, Follow-Up, Safety and Biological Samples, NIDI Study, 2013–2015

Study Area	Indicator	Data Source for Indicator	Results
Enrollment	All eligible M-I pairs at each hospital are screened	Clinical records review	2331 charts screened; 2203 age-eligible infants identified
	Enrollment of 422 M-I pairs in 3 months (3–4 pairs/day/hospital)	Enrollment data (weekly monitoring throughout study)	Enrollment of 456 M-I pairs in 9 months
	100% of M-I pairs have properly documented consent forms	Paper copies of consent forms	Successful consent collection from 100% of pairs
Follow-up	10% LTFU (8–9 pairs lost/visit, 380 pairs complete study)	Enrollment data (weekly monitoring throughout study)	15% LTFU (364 infants completed study)
Safety	0 adverse events and 0 study-related severe adverse events	Documentation and follow up of all adverse events (with standard form)	4 adverse events^a^; 5 severe adverse events, 0 study-related severe adverse events
	Infant and maternal death rates below DHS rates for Bolivia	Documentation and follow up of all deaths	Among infants enrolled in the NIDI study, there were 0 maternal deaths and 5 infant deaths, equivalent to 10.8 per 1000, less than half of Bolivia’s infant mortality rate^b^.
	Monthly reporting of AE/SAE, details of any deaths, reasons for all withdrawals	Documentation of AE/SAE, deaths and reasons for withdrawal using standard forms	100% of withdrawals, deaths, AE/SAE documented
Biological Samples	Successful collection of both infant blood samples from 82% of M-I pairs	Documentation of blood sample collection through survey and laboratory data	Successful collection of first two infant blood samples from 327 infants (75%)
	Successful collection of shedding stool samples from 50% of infants	Documentation of stool collection through survey and laboratory data	Successful collection of shedding stool samples from 75% of infants
	Successful collection of 25% of diarrhea samples (50% loss to non-reporting, collect 50% reported samples)	Documentation of stool collection through survey and laboratory data Comparison to Bolivia DHS data	Successful collection of 61% samples of reported diarrhea cases.

^a^Adverse events included minor bruising and swelling from blood draws

^b^ In Bolivia, there were 23 infant deaths per 1000 post-neonatal infants; 34.7 maternal deaths per 100,000 live births (DHS, 2008)

**Table 3:** Example Monitoring Indicators for Data Collection, NIDI Study, 2013–2015

Study Area	Indicator	Data Source for Indicator^a^	Strategies Taken If Indicator Criteria Not Met (Examples)
Digit Preference (head circumference, weight, and height or length)	Percent of measurements at each digit (0.0–0.9) between 8 and 12%	Infant length, maternal height, and weight data	- Re-training of interviewers - Communication of finding in monthly meetings
Percent Missing Data (age, weight, height or length, and gender)	No missing data	Survey data	- Explanation of importance of complete data collection - Incentives for months with no missing data
Standard Deviation of Z-Scores (WLZ, LAZ, WAZ)	LAZ: 1.10–1.30 WAZ: 1.00–1.20 WLZ: 0.85–1.10[53]	Infant length, maternal height, and weight data	- Communication in monthly meetings - Recognition of interview staff for meeting of goals
Discrepancies in calculated v. reported age (in days)	Discrepancies ≥15 days flagged	Survey data	- Use of calculated age in analysis - Implementing data check of surveys at data entry point
Age range for given visit, number of infants outside specified range	0 infants outside of specified range	Survey data	- Review of protocols for recruitment - Reminder calls to mothers for visits
Referrals for stunting, wasting and those with ongoing diarrhea	100% of infants correctly referred	Survey data; referral forms	- Retraining on referral protocol - Recognizing staff members in monthly meeting for correctly referring study participants

^a^All indicators were monitored on an ongoing basis and presented in a monthly report to Bolivia and Emory University staff

Participant engagement (e.g., recruitment, retention, safety) M&E indicators were determined based on the statistical power needed to answer the primary and secondary study objectives, the estimated loss-to-follow up based on previous pilot studies at the hospitals, estimated non-compliance with sample collection. Rigorous participant safety M&E indicators focused on adverse events (AE) related to the infant blood draws and on infant mortality (a severe adverse event (SAE) not expected to be related to the study) because of the high infant mortality rate in Bolivia [30]. This AE and SAE monitoring was not required by Emory Institutional Review Board or the Bolivian National Bioethics Committee, as this study was not an interventional trial, but was done to ensure that any unforeseen risks to participants would be identified quickly during data collection, so they could be addressed in real time. Biological sample M&E indicators were determined in collaboration with the laboratories that analyzed the resulting samples, focusing on the factors most likely to affect stool or plasma quality and analysis. Anthropometric M&E indicators were developed by the expert anthropometrist (PSS) based on World Health Organization (WHO) best practice [31, 32]. Anthropometric measurements were monitored closely using multiple indicators (Table 3) because of the difficulty of precise and accurate anthropometric data collection in infants and because these measurements directly affected the primary study outcome. Because the study staff were collecting the infants’ anthropometric data for their well-child visits (rather than the hospital nurses), an additional participant safety M&E indicator included the percentage of infants with chronic or acute malnutrition that were identified as such upon anthropometric measurement, to ensure that study participants were appropriately flagged for evaluation by their pediatricians during the well-child visits that followed the study visits.

### Staff training

NIDI staff underwent a 10-day training on the Bolivian health system, interview protocols, informed consent, anthropometry, clinical sample collection and processing, treatment referrals, and AE. An experienced anthropometrist (PSS) conducted anthropometry training using methods developed by the WHO [33, 34]. During the initial training and bi-annual standardizations, staff measured the recumbent length of 10 children under two years old twice, and continued until they produced measurements within 0.5 cm of repeat measurements and of the expert anthropometrist’s measurement in order to optimize precision and accuracy of measurements [33]. Staff were trained in human subjects research ethics using the FHI 360 Research Ethics Training Curriculum (FHI 360, Durham, NC) and passed the corresponding evaluation [35]. Phlebotomy staff at study hospitals were trained on study protocols for blood collection, processing, and cold chain following Centers for Disease Control and Prevention (CDC) guidelines [36]. During training, indicators of participant’s engagement, biological sample and data collection and quality to be measured during the study were shared with staff.

### Participant’s engagement and indicators

#### Enrollment and retention

**Fig. 1:**
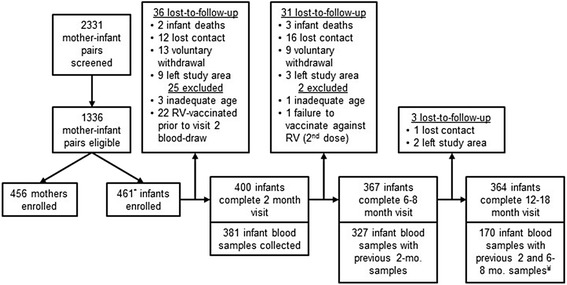
The NIDI Study Enrollment and Loss to Follow-up, 2013–2015. * Five twin pairs were enrolled. ^¥^ The third infant blood draw was at 12–18 months of age due to new funding and new research questions, after many pairs had already completed the final study visit, resulting in a smaller sample size

**Fig. 2:**
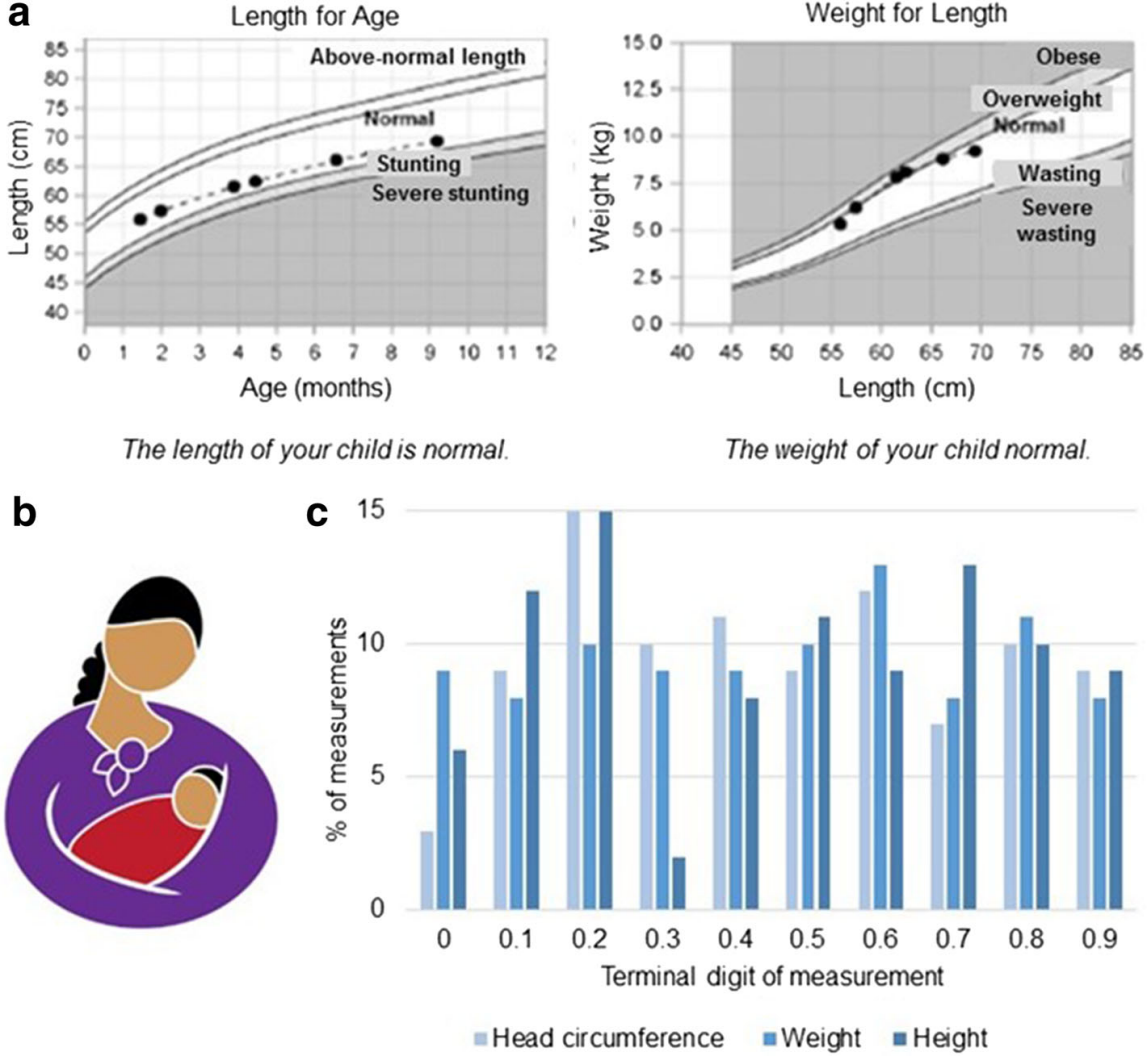
NIDI Study Documents. **a** An example of the growth charts included in the handout mothers were given at the end of the study. See Appendix A for SAS program code used to create the growth chart, NIDIGrowthCurveMacro.sas. **b** The NIDI study logo (pending copyright), featuring an indigenous Aymara mother and infant. The logo was used on study documents, staff clothing and identification cards so that study participants could quickly find and identify staff. **c** An example of a figure from the monthly monitoring and evaluation reports. Identifying any digit preferences in anthropometric measurements was one method used to monitor measurement quality

Potential study participants were identified in the outpatient clinic waiting rooms of Hospital Municipal Corea or Hospital Los Andes. Mothers of young infants were approached, and if interested, their eligibility was assessed via a screening questionnaire. Mothers were eligible if they were ≥15 years, in general good health, the mother of an eligible infant, and were willing to bring their infant to the study hospital for well-child and study visits through one year of age. Infants were eligible if they were ≥14 days old, in general good health, and had not yet been vaccinated with Rotarix®. Infants were ineligible if they had fever, diarrhea, frequent cough, respiratory infection, or hospitalization in the past week, had been diagnosed with a birth defect, chromosomal disorder affecting growth, or immunodeficiency disorder. Eligible mothers were given information sheets and invited to enroll that day or at their next well-child visit (Fig. 1). The NIDI study logo (Fig. 2b) was on all study documents, and staff wore study clothing and photo identification cards bearing the logo so that mothers could easily identify study staff in the hospitals. Mothers were compensated for their time at each visit with food staples (e.g., 1 pound fortified rice). Enrollment and retention indicators included weekly and monthly enrollment numbers and percent lost to follow up by visit.

#### Participant safety

RV vaccination was conducted as part of the Bolivian national vaccination schedule following the blood draw at the second study visit which coincided with the infants’ 2-month well-child visits. The blood draw was considered an intervention in this study. RV vaccination was not a study intervention as it was administered as part of standard well-child care by hospital staff following the Bolivian national vaccination schedule. Thus, vaccination-related AE would have been recorded but not considered study-related AE. Expected related AE were blood draw complications including bruising, infection, or excessive bleeding within 48 h of blood collection. No study-related SAE were expected; expected unrelated SAE included hospitalization, disability, or death. All AE and SAE were reviewed by a pediatrician to determine severity and relation to the study within 48 h. AE and SAE indicators included review and follow-up of all AE and SAE reported by participants, number of study related AE and number and follow-up of study and non-study related SAE.

Mothers were referred to treating physicians for a body-mass index <18.5 kg/m^2^ (or mid-upper arm circumference < 21.0 cm if pregnant) or Hb <14.7 g/dl (Hb < 13.7 g/dl if pregnant) [37]. Infants were referred to treating physicians for moderate or severe undernutrition (LAZ < −2 or WLZ < −2 [26]), anemia (Hb < 10.9 g/dl as per Bolivian national guidelines [38]), for any signs of severe illness, or if mothers reported diarrhea. If mothers reported diarrhea between visits, staff would collect a stool sample, test for RV, enterotoxigenic *E. coli* (ETEC), enteropathogenic *E. coli* (EPEC), enteroaggressive *E. coli* (EAEC), and shared results with the treating physician. Maternal or infant morbidity indictors related to participant safety included referral of each malnutrition case to appropriate physicians for treatment and follow-up of all malnutrition cases at subsequent visits.

### Biological sample collection, quality, and indicators

At each visit, mothers provided their infant’s diaper with stool, if available. Study staff provided a clean diaper and transferred the stool to a sample container and a portion to Cary-Blair transport medium. Samples were stored at 2–8 degrees Centigrade (°C) and transported on ice within 24 h to the Universidad Mayor de San Andrés Instituto de Biología Molecular y Biotecnología (UMSA-IBMB) laboratory for processing. Stool samples were analyzed for RV with the ProSpecT RV Enzyme-linked immunosorbent assay (ELISA) kit (Oxoid, Basingstoke, UK). If no formed stool was available in the soiled diaper, the soiled area of the diaper was eluted in 500 ul of PBS. Stool was also tested for ETEC, EPEC, and EAEC as described in Gonzales et al., 2013 [39]. Stool collection and quality indicators included the percent of samples successfully collected following maternal report of infant diarrhea.

Venous blood was collected from mothers approximately one and 6–8 months postpartum, and from infants at two, 6–8, and 12–18 months of age by the phlebotomy staff at study hospital laboratories (Fig. 1). Blood was collected by trained hospital phlebotomists and processed by study staff using sterile, disposable equipment. Blood was drawn using Safety-Lok™ 23-gauge winged needles and trace-metal-free EDTA Vacutainers® (BD, Franklin Lakes, NJ). An aliquot of whole blood from the needle was analyzed for Hb using the HemoCue® Hb 201+ System (HemoCue AB, Ängelholm, Sweden); anemia was defined using cut-offs adjusted for altitude (3500 m) and pregnancy [40]. The remaining sample was processed to plasma as per Vacutainer® instructions: the vial was inverted 8 times, then centrifuged for 15 min at 1300 relative centrifugal force (RCF) (LW Scientific USA, Lawrenceville, GA) to separate the blood. The plasma supernatant was then transferred to microcentrifuge tubes and centrifuged for 3 min at 2200 RCF (Cole Parmer, Vernon Hills, IL) to remove any remaining precipitates. The supernatant was stored in screw-cap microtubes at 2–8 °C (up to 24 h) before being transported on ice to the UMSA-IBMB laboratory, where the tubes were stored at −70 °C until being transported on dry ice to Emory University laboratories following International Air Transport Association and CDC regulations. There plasma was aliquoted and shipped on dry ice to final analysis locations. Blood quality indicators included percent of samples with hemolysis, storage temperature and storage time.

Plasma was analyzed for RV-specific Immunoglobulins A (IgA) and G (IgG) by Enzyme-linked immunosorbent assay (ELISA) by the Gastroenteritis & Respiratory Viruses Laboratory, Division of Viral Diseases, CDC as described in Moon et al., 2010 [41]. Plasma samples were analyzed for retinol binding protein (RBP), ferritin, soluble transferrin receptor (sTfR), alpha(1)-acid-glycoprotein (AGP), and C-reactive protein (CRP) by the VitMin Laboratory in Willstaett, Germany, as described in Erhardt et al., 2004 [42], and for plasma zinc using inductively-coupled plasma optical emission spectrometry by the Children’s Hospital of Oakland Research Institute Elemental Analysis Facility as described in Engle-Stone et al. 2014 [43]. A subsample (10%) were also tested for retinol; results were used to validate RBP cutoffs for vitamin A deficiency since the molar ratio of retinol-to-RBP is not always 1:1 [44]. All micronutrient biomarkers were adjusted for the effect of inflammation using a novel regression correction approach [45–47] .

### Data collection, quality, and indicators

Vaccination data for the infants was collected from the infants’ clinical vaccination records, provided by the mothers at each study visit and was recorded by study staff as part of each study visit’s data collection. Sociodemographic data were collected at the first and last visit, morbidity and clinical data at all visits. Mothers were given a wall calendar and stickers of images indicating morbidities (diarrhea, fever, cough, hospitalization) as a memory aid to record infant morbidities. Mothers were asked about infant feeding practices, including breastfeeding. Infant nutritional status was determined by LAZ, WLZ, and head circumference at each visit by trained interviewers using standardized wooden boards to measure supine length (Shorrboards, Olney, MD) and an electronic M-I floor scale to measure weight (SECA scales, Hanover, MD). Measurements were placed in the infant’s clinical chart for their corresponding well-child visit, and mothers were given a handout at the end of the study documenting their child’s growth throughout the study (Fig. 2a). The SAS program code used to create the growth chart (NIDI_GrowthCurveMacro.sas) is provided as an Additional file 2. Maternal height and weight were collected at each visit to calculate body mass index. Data were collected on paper forms and double-data entered using the web-based research electronic data capture (REDCap) system hosted at Emory University (UL1 TR000421) [48]. Discrepancies between entries were reconciled within a week. Any biologically implausible data points were corrected or set to a missing value. Sample processing and cold chain data were double-entered and reconciled in Microsoft Excel (Seattle, WA).

Data collection and quality indicators included digit preference for anthropometry measures (height, weight and length), standard deviations of weight-for-length, length-for-age and weight-for-age z-scores and percent missing anthropometric data for each visit.

### Reporting and application of M&E indicators

Emory University staff created monthly reports of monitoring indicators including measures of participant retention, biological sample collection (e.g., hemolysis, storage temperature, and storage time for blood samples), and data quality. LTFU and reasons for withdrawal were presented by visit. For anthropometry data quality indicators (Table 3), digit preferences in measurements were visualized in a graph (Fig. 2c). Reports were shared with study staff during monthly meetings, strategies were developed to improve data quality, goals were set for the next month, and rewards were given for meeting quality targets. All study components were monitored in-person by principal investigators or project managers 2–3 times a year.

## Results

While indicator targets for participant enrollment were met, the number of infants lost to follow-up was greater than expected. Of 2331 M-I pairs initially screened, 1336 infants were eligible, and 456 mothers and 461 infants (including 5 twins) were enrolled in the study June 2013–April 2014, exceeding the target of 422 M-I pairs. Six percent (27/461) of infants were excluded as ineligible, primarily due to having received the RV vaccine prior to the first infant blood-draw. Fifteen percent (70/461) of infants were lost to follow-up, which did not meet the target of <10% LTFU (Fig. 1). However, 91% (364/400) of infants that completed the first two visits completed the study. In total, 364 infants completed the study through the final visit.

There were four study-related AE (all minor swelling and bruising related to the blood draw), no study-related SAE, and five infant deaths (SAE) unrelated to the study. SAE included hypoxic-ischemic encephalopathy (age 4 months), pneumonia (two at ages 2 and 3 months), diarrhea (age 2 months), and sudden infant death syndrome (age 3 months). After review of death certificates and conducting verbal autopsies when necessary, a pediatrician determined each death was unrelated to the study due to its medical cause. This mortality rate of infants in the study, equivalent to 10.8 per 1000 enrolled infants, is less than half the infant mortality rate in Bolivia (23 per 1000 post-neonatal infants) [27].

Biological sample indicator targets were met for stool sample collection but not blood sample collection (Table 2). There were 389 successful infant blood draws at 1–2 months (baseline), but only 333 (95% of the required 350 blood sample size) also had a 6–8 month sample (Fig. 1). Hemolysis occurred frequently during blood draws, despite proper collection and processing technique, but did not affect sample analysis. Stool samples to assess post-vaccination RV viral shedding were collected from 75% of infants, while 61% of reported diarrhea cases had associated stool samples collected.

Anthropometry data quality indicators were monitored throughout the study (Table 3). The medians of monthly standard deviations for weight-for-length, length-for-age, and weight-for-age z-scores were 1.01, 0.98, and 1.03, respectively; close to the expected value of 1.0 for a reference distribution. Standard deviations for z-scores varied month-to-month, but never reached the WHO thresholds for measurement error or incorrect age reporting [49]. However, only 61% of measurements indicating stunting and only 56% of measurements indicating wasting resulted in an immediate referral for treatment. Referral practices improved during the second year of data collection: 87% of measurements indicating stunting and 91% of measurements indicating wasting resulted in an immediate referral. Throughout the study, these indicators were presented to study staff each month to encourage improvement and follow-up. Thus, all stunted and wasted children who had not initially been referred were followed-up to ensure they received treatment.

## Discussion

This paper describes the data and participant safety M&E framework of an observational cohort study of mothers and their infants and challenges encountered in the implementation of this study in El Alto, Bolivia. The M&E framework allowed staff to respond both to predicted and unforeseen challenges in study implementation.

The study met its enrollment objective (450 M-I pairs). However, it collected 95% of the required first two infant blood samples from 350 infants which meant it did not meet its sample size objectives (350 infants), although 364 infants completed the final study visit. Follow-up was hindered by unexpected and rapid policy changes to the Bolivian health system in December 2013, which shifted well-child visits to primary care clinics during data collection [50, 51]. Though participants could continue well-child visits at the study hospital, conflicting national messages and the convenience of primary care clinics reduced follow-up.

Reported AE were minor (local bruising and swelling related to the blood draw). All infant deaths were determined to be unrelated to the study after verbal autopsy and review of the death certificate by the study pediatrician. Though the target for this indicator was no SAE during the course of the study, infant mortality in the study was less than half of the post-neonatal infant mortality rate in Bolivia [27]. This could be due to differences between the national population and the populations attending the two study hospitals.

The study collected samples from only 18% of the predicted diarrhea case count, but collected a sample 61% of the time when diarrhea was reported. As the monthly monitoring indicator was the percent of reported diarrhea that resulted in sample collection, no changes were made in study protocol to collect more samples. Diarrhea may have been under-reported, but it is also possible that the population simply had fewer cases of diarrhea during the first year of life than were predicted based on pilot data. Post-RV vaccination stool samples collection (75%) exceeded the goal of 50% of enrolled infants, likely due to the convenience of the home visit(s) for sample collection.

Anthropometry data quality was extremely good, likely due to extensive training, standardization, and monitoring efforts. However, many children were not immediately referred for treatment when they were measured as stunted or wasted. Each month, this indicator was measured and presented to study staff to encourage improvement and follow-up. Thus, all stunted and wasted children who had not initially been referred were followed-up to ensure they received treatment. Poor referral practices were likely the result of failure to identify stunting and wasted infants using the WHO growth charts, especially in the youngest infants and early in the study, when staff burden was highest. In addition, study staff stopped referring children measured as stunted at repeated visits, as they were already being monitored by their physicians.

The monitoring plan, based on the M&E framework indicators, successfully allowed course-correction and quality improvement throughout the study. Well-trained local staff, a clear protocol for AE, and a comprehensive data management plan facilitated the success of the study. Having well-trained staff who spoke local languages and understood the political and cultural landscape allowed the study to continue despite local political unrest and health policy changes. Preparation for, and management of, AE took considerable time and resources. Although this was an observational study, this effort was warranted given cultural sensitivity to blood collection and the vulnerable population (infants as young as two months). Having a comprehensive data management plan allowed for high quality data control. Monthly monitoring meetings in which the M&E framework indicators were reviewed and discussed with in-country staff maintained a sense of accountability and ownership, and allowed for open discussion of study challenges and potential solutions.

Some aspects of development, implementation and adaptation of the M&E framework could be improved in future studies. In the development of the framework, indicators for staff satisfaction and retention were not considered, although staff data collection burden was monitored. As training and management of local staff took considerable time and resources, monitoring of staff satisfaction may have allowed earlier course correction in this area. In the second year of the study, staff satisfaction surveys were implemented, which allowed in-country and Emory University project managers to improve staff satisfaction and provide additional resources to in-country staff as needed. In the implementation of the framework, specific aspects of how indicators would be analyzed and communicated to all study team members were not considered until data collection was underway. Including a detailed communication structure in the framework would have ensured uniformity and continuity of communication from the beginning. Finally, the study design and M&E framework were not revised following an unexpected and rapid policy change in the Bolivian health system that influenced enrollment and retention [50, 51]. Allowing the study design and M&E framework to adapt to external features such as changes in the health system would have improved implementation.

## Conclusions

The data and participant safety M&E framework described here and lessons learned from this study can be applied to other observational studies to improve the quality and comparability of data. The data and current [46, 52] and forthcoming publications will advance the base of knowledge on oral vaccine underperformance and infant undernutrition in developing countries.

## Additional files.

Additional file 1: NIDI_Study_Questionnaire_Visit_1.pdf. NIDI Study Questionnaire - visit one. NIDI study questionnaire for visit one – baseline data collection. Includes informed consent. Note: the questionnaire was developed in Spanish and is presented here in Spanish (SAS 7 kb)

Additional file 2: NIDI_GrowthCurveMacro.sas. SAS Program Code for Infant Growth Chart. SAS Macro to create graphs that show individual child growth over time compared to a WHO reference (PDF 882 kb)

## Abbreviations

AE: Adverse events
AGP: Alpha(1)-acid-glycoprotein
CRP: C-reactive protein
EAEC: Enteroaggressive *E. coli*
ELISA: Enzyme-linked immunosorbent assay
EPEC: Enteropathegenic *E. coli*
ETEC: Enterotoxigenic *E. coli*
Hb: Hemoglobin
IgA: Immunoglobulin A
IgG: Immunoglobulin G
LAZ: Length-for-age z-score
LTFU: Loss to follow-up
M&E: Monitoring and evaluation
M-I: Mother-infant
NIDI: Nutrition, Immunology, and Diarrheal Illness or Nutrición, Inmunología y Diarrea Infantil
RBP: Retinol binding protein
RCF: Relative centrifugal force
REDCap: Research electronic data capture
RV: Rotavirus
SAE: Severe adverse events
sTfR: Soluble transferrin receptor
UMSA-IBMB: Universidad Mayor de San Andrés Instituto de Biología Molecular y Biotecnología
WLZ: Weight-for-length z-score
