# Supplementary material for: Using a monitoring and evaluation framework to improve study efficiency and quality during a prospective cohort study in infants receiving rotavirus vaccination in El Alto, Bolivia: the Infant Nutrition, Inflammation, and Diarrheal Illness (NIDI) study
Source: BMC Public Health. 2017 Nov 28;17:911. doi: 10.1186/s12889-017-4904-5 (PMC5706310; doi:10.1186/s12889-017-4904-5)
Supplement: Supplementary file 2 — NIDI_GrowthCurveMacro.sas. SAS Program Code for Infant Growth Chart. SAS Macro to create graphs that show individual child growth over time compared to a WHO reference (PDF 882 kb) [file 12889_2017_4904_MOESM2_ESM.pdf]

VISITA 1 ID del estudio: ________

Carta de Consentimiento Informado para Participación en el Estudio

Yo, ________________________________ autorizo la participación mía y la de mi hijo(a) _____________________________________ en el estudio titulado:

"Evaluación de Nutrición, Inmunología, y Diarrea Infantil (NIDI),"(Aprobado por el Comité de Ética Nacional de Bolivia).

Este estudio se desarrollara en la Ciudad de El Alto y está coordinado por la Universidad de Emory en EEUU, la UMSA, y la ONG CAIA, y los hospitales Materno Infantil "Los Andes" y Municipal Modelo "Corea".

Se me ha informado que:

- El propósito del estudio es entender si la nutrición del niño ayuda a la vacuna contra el rotavirus.
- Mi participación consistirá en dar permiso para que se haga seguimiento de mi hijo(a) y mi persona durante 12 meses.
- Durante dicho periodo, y mediante visitas de estudio, se tomaran medidas como talla y peso, datos clínicos del expediente, del hogar, educación y muestra de sangre (25 gotas) en dos ocasiones, así como la recolección de la caquita de mi hijo(a).
- En caso de que el técnico no pueda obtener la muestra al segundo intento se me dará la opción de que se tome sangre mediante un piquete del dedo de mi mano y del talón en el caso de mi hijo(a).

Declaro que se me ha informado ampliamente que mi participación es completamente voluntaria.

Como beneficios de este estudio, tanto mi hijo(a) como yo tendremos:

- El análisis gratuito de sangre para saber si tenemos anemia y como está nuestro estado nutricional.
- El análisis gratuito de la caquita de mi hijo(a) para conocer que microbio es el que está causando la diarrea.

Además se me dio a conocer que este estudio es de bajo riesgo para mí o para mi hijo(a). Los posibles riesgos son: dolor, moretón, o posible hinchazón durante o después del pinchazo de la muestra de sangre. Entiendo que mi identidad y la de mi hijo serán protegidas, y que la información se manejara de manera confidencial.

Entiendo que soy libre de retirarme así como a mi hijo(a) del estudio en cualquier momento, sin que ello afecte la atención médica que por derecho nos corresponde. Entiendo que si me retiro, toda mi información y las muestras obtenidas serán parte del estudio.

Se me han dado números telefónicos a los cuales me puedo comunicar en caso de emergencia y/o dudas y preguntas relacionadas con el estudio:

Coordinadora del estudio:
Correo: coordinaciongral.nidi@caiabolivia.org
Teléfono coordinadora: 71523889
Teléfono encuestadoras de hospital: ______________________________

Por tanto autorizo a que mi hijo(a) participe del presente estudio.

Nombre Participante ____

C.I. o número de historia clínica ____

Firma ____

Fecha ____

VISITA 1 ID del estudio: _________

Por tanto autorizo a que mi hija y nieto(a) participe del presente estudio.

Nombre del padre** ____

C.I. ____

Firma ____

Fecha ____

*** Necesario si la participante mama es menor de edad Y vive con sus padres (abuelos del niño)*

Nombre del testigo** ____

Relación con participante ____

C.I. ____

Firma ____

Fecha ____

Nombre del responsable de la
Obtención del consentimiento: ____

C.I. ____

Firma ____

Fecha ____

Lugar donde se obtuvo ____

VISITA 1 ID del estudio: _________

Página de Cubierta

Estudio NIDI: Nutrición, inmunología, y diarrea infantil en Bolivia

Número de identificación del estudio	___ ___ ___
¿El niño tiene un mellizo o trillizo también en el estudio?	○ No<br>○ Gemelo/Mellizo<br>○ Trillizo<br>Los mellizos y trillizos comparten el mismo número de identificación del estudio. Se distinguen por la letra asociada con el niño (B, G, T)<br>En caso de mellizo o trillizo, obtenga las encuestas especificadas, incluyendo otra página de cubierta
Número visita	○ 1ª visita<br>○ 2ª visita<br>○ 3ª visita<br>○ 4ª visita<br>○ 5ª visita<br>○ 6ª visita<br>○ 7ª visita<br>○ 8ª visita<br>○ no es una visita planificada
Hospital	○ Los Andes<br>○ Corea
Fecha de entrevista o visita:<br>___ ___- ___ ___- ___ ___ ___ ___<br>**(**dd mm aaaa)	Hora de comienzo de entrevista<br>___ ___ : ___ ___<br>(HH:MM)
¿Dónde le hablaron sobre el estudio?<br>○ Hospital<br>○ Centro de salud<br>○ Otro_____________	¿Quién le hablo sobre el estudio NIDI?<br>○ Encuestadora **NIDI**<br>○ Personal de salud<br>○ Otro________________
¿Obtuvo el consentimiento informado?	○ No<br>○ Sí
¿Cuál es la fecha de nacimiento del bebé? Verifique con el carnet	___ ___—___ ___—___ ___ ___ ___<br>(dd—mm—aa)<br>99-99-9999 La madre no sabe
¿Es él bebe niño o niña?	○ Niño<br>○ Niña
¿Cuál es la fecha de nacimiento de la madre? Verifique con el carnet	___ ___—___ ___—___ ___ ___ ___<br>(dd—mm—aa)<br>99-99-9999 La madre no sabe
Hora fin de entrevista	___ ___ : ___ ___<br>(HH:MM)
Fecha de próxima visita:	___ ___- ___ ___- ___ ___ ___ ___<br>(dd mm aaaa)
Muestra de heces<br><br>___ ___ - _______- ___ ___ ___ ___<br>dd mm aaaaa	○ RV Elisa (visita 1,2,3)<br>○ Asintomático<br>○ Diarrea

**Firma del personal del estudio**	Criticado en el campo por iniciales	Digitado en REDCap por iniciales
Nombre Entrevistadora<br>______________________	Iniciales<br>___ ___	Iniciales<br>___ ___

VISITA 1

ID del estudio: _________

___ ___- ___ ___- ___ ___ ___ ___<br>(dd mm aaaa)	___ ___- ___ ___- ___ ___ ___ ___<br>(dd mm aaaa)	___ ___- ___ ___- ___ ___ ___ _<br>(dd mm aaaa)

Formulario de Identificación de Residencia

ID del Estudio__ __ __

Fecha de Autorización__ __/__ __/__ __ __ __

Nombre de la Madre: ____________________________________

Nombre del bebe:__________________________

Número de Teléfono____ __ ____ __ __ __

Dirección (dada en el momento de autorización):

____

____

¿En qué zona se encuentra su casa?____

¿Quién más vive en esta casa?

Nombre: _______________________ Relación: _______________Teléfono: ____ __ ____ __ __ __

Nombre: _______________________ Relación: ______________ Teléfono: ____ __ ____ __ __ __

Nombre:_______________________ Relación : ______________ Teléfono: ____ __ ____ __ __ __

Por favor pídele a la madre que dibuje la ubicación de su residencia:

Información adicional que puede ayudar al personal del estudio a encontrar su residencia (algún punto de referencia obvio o nombre de calle principal):

____

____

____

SOLO PARA EL USO DEL PERSONAL (Danny – Mary)

- ☐ La residencia de la participante fue identificada y confirmada
- ☐ La residencia de la participante fue identificada pero no confirmada
- ☐ La residencia de la participante no fue identificada ni confirmada

VISITA 1 ID del estudio: _________

Coordenadas de GPS: (N/S □□□°, □□ .□□□) (E/W □□□°, □□ .□□□)

Fecha: __ __/__ __/__ __ __ __Nombre del Personal: ________________________________

VISITA 1

ID del estudio: _________

Antropometría (Madre)

Medidas Antropométricas de la Madre

Nro.	*Pregunta*	*Respuesta con Códigos*	*Patrón de saltos*
1.	Talla de la madre (cm)<br><br>También marque la talla en la tarjeta de resultados NIDI de la madre	☐☐☐ . ☐ cm<br><br>999.9 No medida	
2.	Peso de la madre (kg)<br><br>**Sin zapatos, sin chompa, sin pollera, con la bata del proyecto**<br><br>También marque el peso en la tarjeta de resultados NIDI de la madre	☐☐ . ☐ kg<br><br>99.9 No medida	
3.	¿Está embarazada la mama?	○ Si<br>○ No<br>○ NS/NR	En caso de si<br>→ 5
4.	Índice Masa Corporal de la madre	☐☐ . ☐ kg/m²	
5.	Perímetro braquial de la madre (cm)<br>Solo en caso de mujer embarazada – También marque el perímetro braquial en la tarjeta de resultados NIDI de la madre	☐☐ . ☐ cm<br><br>99.9 No medida<br><br>○ No aplica porque no está embarazada	
6.	En caso de que la madre está utilizando ropa, indique cuáles son<br><br>Indique que estaba llevando la mamá, y la cantidad de cada prenda que llevaba. Marque también el material de cada prenda.	○ Bata NIDI<br>○ Blusas/Polera_____<br>○ Chompas (de lana ligera, gruesa, o de polar) _______<br>○ Mallas (corta o larga) (tela, seda, o lana) _______<br>○ Fajas_______<br>○ Polleras_______<br>○ Centros (tela, seda, o lana) _______	○ Pantalones _______<br>○ Jeans_______<br>○ Buzos_______<br>○ Medias_______<br>○ Gorra / Sombrero / Gorro _______<br>○ Guantes ______<br>○ Otro______________<br>○ NS/NR
7.	¿Hubo alguna injerencia con las medidas?	○ No<br>○ Sí<br>○ NS/NR<br>Observaciones: ____	
	Iniciales de Antropometrista:<br><br>_______________________	Iniciales de Asistente:<br><br>_____________________________	

Antropometría (Niño)

Medidas Antropométricas del bebé

Nro.	*Pregunta*	*Respuesta con Códigos*	*Patrón de saltos*
8.	Obtenga el formulario ID estudio y/o Carnet Infantil y anote la fecha del nacimiento del niño.<br>También marque la fecha de nacimiento en la tarjeta de resultados NIDI del bebe	__ ___-___ ___-___ ___ ___ ___<br>(dd– mm -aa)<br>99-99-9999 La madre no sabe	
9.	Usando la fecha del nacimiento del niño, anote la edad del niño en días y meses	○ ________meses<br>○ ________días	
10.	Anote el género del niño desde el formulario ID estudio o Carnet Infantil.	○ Niño<br>○ Niña	
11.	¿Está sucio ahora el pañal del bebé?	○ No<br>○ Sí	En caso de no →13
12.	¿Nos podría dar el pañal sucio del bebé para que le podamos hacer una prueba para ver si tiene gérmenes/virus? A cambio, le daré un pañal nuevo.<br>En caso de sí, pedirle el pañal al fin de la entrevista y complete página 1 del “Formulario de Recolección de Heces”	○ No<br>○ Sí	
13.	Peso del niño(a) (kg)<br>**Sin pañal**<br>También marque el peso	☐☐.☐kg<br>99.9 No medida	
14.	Longitud del niño (a)(cm)<br>También marque el longitud en la tarjeta de resultados NIDI del niño	☐☐☐ . ☐cm<br>999.9 No medida	
15.	Perímetro cefálico del niño(a) (cm)	☐☐.☐cm<br>99.9 No medida	
16.	Examine el área de la tibia o cerca del tobillo, para la presencia de edema en ambas piernas ¿Él bebe tiene edema bilateral?	○ No<br>○ Sí **(llene boleta de referencia)**<br>○ No sabe	

17.	¿Hubo alguna injerencia con las medidas o durante el examen?<br>Describe y enumere la ropa del niño y marque cualquier otra injerencia	○ No<br><br>○ Sí<br>Observaciones (ropa):_________________________________

Utilice las gráficas de crecimiento laminadas para determinar si hay "baja talla para la edad" o "bajo peso para la talla" en los niños.

Recuerde: si el punto está igual o menos de 2 desviaciones estándares, recibirá la clasificación de "baja talla para la edad" o "bajo peso para la talla." Si necesita más información, revise el Manual de Referencia.

18.	En base a las tablas de referencia, ¿el niño tiene baja talla para la edad (=<-2 puntaje Z)?	○ No<br>○ Sí **(llene boleta de referencia)**	
19.	En base a las tablas de referencia, ¿el niño tiene bajo peso para la talla (=<-2 puntaje Z)?	○ No<br>○ Sí **(llene boleta de referencia)**	
	Iniciales de Antropometrista:<br>________________________	Iniciales de Asistente:<br>________________________________	

VISITA 1 ID del estudio: _________

Encuesta: Sociodemográfica (Madre)

Información Personal Ahora, le voy a hacer algunas preguntas sobre usted, su hogar y su familia. Esta información será utilizada para mejorar nuestro conocimiento de los participantes del estudio.

Nro	*Pregunta*	*Respuesta con Códigos*	*Patrón de saltos*
1.	¿Se considera perteneciente a algún grupo indígena?	○ No<br>○ Sí<br>○ NS/NR	En caso de no →3
2.	¿A qué grupo indígena se considera usted descendiente?	○ Quechua<br>○ Aymara<br>○ Guaraní<br>○ Otro _____________<br>○ NS/NR	
3.	¿Cuál es el nivel más alto de instrucción o curso que aprobó?	○ Sin educación<br>○ Primaria incompleta<br>○ Primaria completa<br>○ Secundaria incompleta<br>○ Secundaria completa<br>○ Superior Incompleta<br>○ Superior y más<br>○ NS/NR	
4.	¿Cuál es su estado civil?	○ Casada y vive junto con su esposo<br>○ Concubinato<br>○ Separada/Divorciada<br>○ No tiene una pareja<br>○ NS/NR	
5.	¿Cuantas personas viven en su hogar?	_______________ (número de personas)<br>9999 La madre no sabe	

Información sobre el Trabajo Con su permiso, le voy a preguntar acerca de su trabajo.

Nro.	*Pregunta*	*Respuesta con Códigos*	*Patrón de saltos*
6.	¿Usted tiene un trabajo?	○ No<br>○ Sí<br>○ NS/NR	En caso de no →8
7.	¿Qué tipo de trabajo tiene?	○ Profesional<br>○ Oficinista<br>○ Ventas y servicios (en una tienda)<br>○ Vendedor Ambulante<br>○ Manual calificado (técnico)<br>○ Manual no calificado<br>○ Servicio doméstico<br>○ Agricultura<br>○ Otro_________	

		○ NS/NR	

Información sobre su hogar Ahora voy a hacerle algunas preguntas sobre su hogar.

Nro.	*Pregunta*	*Respuesta con Códigos*	*Patrón de salteos*
8.	¿Usted vive en El Alto?	○ No<br>○ Sí	Si no →10
9.	¿Hace cuánto tiempo vive en El Alto?<br>Selecciona solamente una unidad de tiempo que corresponde con el número	_________ (#)<br>9999 La madre no sabe<br>○ meses<br>○ años	
10.	¿Tiene en su hogar los siguientes elementos?<br>**Lea las opciones listadas en voz alta.Marque todas que apliquen.**	○ A, Radio o equipo de música _______ (#)<br>9999 La madre no sabe<br>○ B, Televisor<br>○ C, Refrigerador<br>○ D, Bicicletas<br>○ E, Motocicletas<br>○ F, Vehículo automotor (para uso personal)<br>○ G, Teléfono fijo _____________(#)<br>9999 La madre no sabe<br>○ H, Teléfono celular ______________(#)<br>9999 La madre no sabe<br>○ I, Computadores<br>○ J, Servicio de internet en el hogar<br>○ K, Bomba eléctrica de agua<br>○ NS/NR	
11.	¿Cuál es la fuente (procedencia) principal de abastecimiento de agua que utilizan los miembros de su hogar para beber y cocinar?	○ Por cañería dentro de la vivienda (cocina)<br>○ Por cañería fuera de la vivienda pero dentro del edificio, lote o terreno (patio)<br>○ Por cañería fuera del lote/terreno (pileta publica)<br>○ Pozo o noria con bomba<br>○ Pozo o noria sin bomba<br>○ Lago, laguna o curiche<br>○ Rio, vertiente o acequia<br>○ Vecinos<br>○ Carro repartidor (aguatero)<br>○ Otro: ______________<br>○ NS/NR	
12.	¿Durante las últimas dos semanas, con qué frecuencia ha estado disponible el agua de esta fuente?	○ Todo el tiempo<br>○ Unas pocas horas diarias<br>○ Lunes a viernes, pero no los sáb., dom.<br>○ Algunos días de la semana<br>○ Menos frecuentemente<br>○ NS/NR	

13.	¿Usted hace algún tratamiento al agua para beber?	○ No<br>○ Sí<br>○ NS/NR	En caso deno o no sé→15
14.	¿Cómo trata su agua, o que tratamiento hace con su agua?<br>**No lea las opciones listadas en voz alta. Marque todas que apliquen.**	○ Hierve<br>○ La cloran<br>○ Filtra con tela<br>○ Usa filtro (cerámica/ arena/ otro)<br>○ SODIS (purifican con luz solar)<br>○ La deja reposar<br>○ Otro ____________<br>○ NS/NR	
15.	¿Cuál es el **principal** combustible que utiliza para cocinar?	○ Leña<br>○ Guano /Bosta o Taquia<br>○ Kerosene<br>○ Gas porGarrafa<br>○ Gas domiciliario<br>○ Electricidad<br>○ No utiliza<br>○ Otro _____________<br>○ NS/NR	
16.	¿Tiene un servicio sanitario (ej. baño o letrina)?	○ No<br>○ Sí<br>○ NS/NR	En caso de no →19
17.	¿El uso del servicio sanitario es privado del hogar o compartido con otros hogares?	○ Privado del hogar<br>○ Compartido con otros hogares<br>○ NS/NR	
18.	¿El desagüe del servicio sanitario se realiza por…….?	○ Alcantarillado<br>○ Cámara séptica<br>○ Pozo ciego<br>○ A la superficie (calle/quebrada/rio)<br>○ Otro _____________<br>○ NS/NR	
19.	¿Cuál es el material más utilizado en los pisos de su vivienda?<br>Especifique que la pregunta se refiere al terminado del piso	○ Tierra<br>○ Tablón de madera<br>○ Machihembre/Parquet<br>○ Alfombra/Tapizón<br>○ Cemento<br>○ Mosaico/Baldosa/Cerámica<br>○ Ladrillo<br>○ Otro _____________<br>○ NS/NR	
20.	¿Cuál es el material de construcción más utilizado en las paredes de su vivienda?	○ Ladrillo/Bloque de cemento/hormigón<br>○ Adobe/tapial<br>○ Piedra<br>○ Madera<br>○ Otro _____________	

		○ NS/NR	

21.	¿Cuál es el material más utilizado en los techos de su vivienda?	○ Calamina/plancha<br>○ Teja (Cemento/arcilla/ fibrocemento)<br>○ Losa de hormigón armado<br>○ Otro: _____________<br>○ NS/NR	
22.	¿Cómo eliminan habitualmente la basura en su hogar?	○ Alcaldía municipal (Empresa contratada por la alcaldía)<br>○ Otra empresa privada o persona<br>○ La queman<br>○ La tiran al patio<br>○ La tiran a la calle/quebrada/rio<br>○ La ponen en un contenedor que está a unas cuadras<br>○ Otro ____________<br>○ NS/NR	
23.	¿Tiene un cuarto sólo para cocinar?	○ No<br>○ Sí	
24.	¿Cuántos cuartos o habitaciones ocupa su hogar, sin contar el baño y cocina?	____________ (número de cuartos)<br>9999 La madre no sabe	
25.	De estos cuartos o habitaciones, ¿cuántos se utilizan para dormir?	____________ (número de cuartos)<br>9999 La madre no sabe	
26.	¿Cuántas personas duermen en cada dormitorio?	____________ (número de personas / dormitorio)<br>9999 La madre no sabe	

VISITA 1

ID del estudio: _________

Encuesta: Datos Clínicos (Madre)

Historia Clínica de la Madre

Carnet maternal: Pregunte a la madre por su carnet perinatal. Primero, trate de contestar las siguientes preguntas por el carnet. Si la información no está disponible, pregunte a la madre.

Nro.	*Pregunta*	*Respuesta con Códigos*	*Patrón de saltos*
1.	**[Primero del carnet]** ¿Cuántos embarazos ha tenido la madre? Incluyendo el actual<br><br>Anote la fuente de la información.	_______________ # de gestas<br>9 9 9 9 La madre no sabe<br>*Fuente:*<br>○ Del carnet<br>○ De la madre	
2.	¿Cuántos partos ha tenido, incluidos cesáreas?<br><br>Anote la fuente de la información.	_______________ # de partos<br>9 9 9 9 La madre no sabe<br>*Fuente:*<br>○ Del carnet<br>○ De la madre	
3.	¿Cuántos partos de nacidos vivos ha tenido?<br><br>Anote la fuente de la información.	___________ # de partos de nacidos vivos<br>9 9 9 9 La madre no sabe<br>*Fuente:*<br>○ Del carnet<br>○ De la madre	
4.	¿Hizo control prenatal durante este embarazo?<br><br>Anote la fuente de la información.	○ No<br>○ Sí<br>*Fuente:*<br>○ Del carnet<br>○ De la madre	En caso de NO →7
5.	¿En semanas, cuándo fue la primera visita de control prenatal durante este embarazo?<br><br>Note la fuente de la información.	___________(semanas)<br>9 9 9 9 La madre no sabe<br><br>*Fuente:*<br>○ Del carnet<br>○ De la madre	
6.	¿Cuántas visitas de control prenatal hizo en total durante este embarazo?<br><br>Anote la fuente de la información.	_______________ # de visitas<br>9 9 9 9 La madre no sabe<br><br>*Fuente:*<br>○ Del carnet<br>○ De la madre	
7.	¿Alguna vez le ha diagnosticado con hipertensión?<br><br>Anote la fuente de la información.	○ No<br>○ Sí<br>○ NS/NR<br>*Fuente:*	

		○ Del carnet<br>○ De la madre	
8.	¿Alguna vez le ha diagnosticado con diabetes?<br><br>Anote la fuente de la información.	○ No<br>○ Sí, cualquier tipo<br>○ NS/NR<br>*Fuente:*<br>○ Del carnet<br>○ De la madre	
9.	¿Le ha diagnosticado con anemia en algún momento durante este último embarazo? Anote la fuente de la información.<br><br>Si cualquier campo de "Anemia" está marcado con "Si" en el carnet, marque Si	○ No<br>○ Sí<br>○ NS/NR<br>*Fuente:*<br>○ Del carnet<br>○ De la madre	
10.	¿Cual fue la edad gestacional al parto?<br><br>Anote la fuente de la información.	_________ (semanas) y ________ (días)<br><br>9 9 y 9 9 La madre no sabe<br><br>Calculada por:<br>○ Medición del fondo uterino<br>○ Ecografía<br>○ Memoria de la madre<br>○ La fecha de ultima regla<br>○ Por examen físico del recién nacido<br>*Fuente:*<br>○ Del carnet<br>○ De la madre	
11.	¿Qué tipo de parto fue?<br><br>Anote la fuente de la información.	○ Espontanea (vaginal)<br>○ Cesárea<br>*Fuente:*<br>○ Del carnet<br>○ De la madre	

Peso del bebé al nacer

12.	¿Cuántos kilos pesó el bebé al nacer?<br><br>Anote la fuente de la información.	☐☐☐☐ gr<br><br>9 9 9 9 La madre no sabe<br>*Fuente:*<br>○ Del carnet<br>○ De la madre	En caso del carnet→14
13.	¿Cuántos días después del nacimiento fue tomado este peso?	__ __<br>(Marque "00" si fue medido al nacer)<br>9 9 9 9 La madre no sabe	

VISITA 1

ID del estudio: _________

Historia Clínica de la Madre

14.	¿Qué edad tienen sus hijos vivos (años, meses y días)?<br><br>Incluir todos los hijos empezando por el mayor.	Hijo 1______Hijo 6______<br>Hijo 2______Hijo 7 ______<br>Hijo 3 ______Hijo 8 ______<br>Hijo 4 ______Hijo 9 ______<br>Hijo 5 ______Hijo 10 ______<br>9999 La madre no sabe	
15.	¿Dónde nació el bebé?<br><br>Especifique	○ En este hospital<br>○ En otro hospital, público ______________________<br>○ En otro hospital, privado ______________________<br>○ En un centro de salud ______________________<br>○ En su casa<br>○ Otro:______________<br>9999 La madre no sabe	
16.	¿Hubo alguna complicación durante el parto?	○ No<br>○ Sí<br>○ NS/NR	En caso de no→18
17.	¿Qué tipo de complicaciones hubo?<br><br>No lea las opciones listadas en voz alta.<br><br>Marque todas que aplican.	○ Sangrado excesivo<br>○ Trabajo prolongado del parto<br>○ Sufrimiento fetal<br>○ Infección o fiebre materna<br>○ Otra ___________________<br>○ NS/NR	
18.	¿Usted Fuma?	○ No<br>○ Sí<br>○ Ocasionalmente<br>○ NS/NR	En caso de no o no sabe →20
19.	¿Cuántos cigarrillos fuma al día?	○ < ½ paquete = <10 cigarrillos<br>○ ½-1 paquete = 10-19 cigarrillos<br>○ 1-2 paquetes=20-40 cigarrillos<br>○ 2 paquetes+ = 41 cigarrillos+<br>○ NS/NR	

20.	¿Normalmente, toma bebidas alcohólicas?<br><br>**Si no, indague:** ¿No toma o toma a veces?	○ No, nunca<br>○ Sí, a veces<br>○ Sí, frecuentemente<br>○ NS/NR	En caso de no o se negó →23
21.	Durante los ÚLTIMOS tres meses de su embarazo, ¿tomó bebidas alcohólicas?	○ No<br>○ Sí<br>○ NS/NR	En caso de no o se negó →23
22.	Durante los ÚLTIMOS tres meses de su embarazo, ¿Cuántas bebidas alcohólicas tomaba por semana?<br><br>Cuando dice bebida se refiera a:<br>310 ml para la cerveza paceña<br>135 ml para licores<br><br>OJO (1 botella tiene 4 vasos, multiplicar de acuerdo a la respuesta)	○ Menos de una bebida<br>○ 1-3 vasos<br>○ 4-6 vasos<br>○ 7-13 vasos<br>○ 14+ vasos<br>○ Otro ______________<br>○ NS/NR	
23.	¿En los últimos 6 meses, ha tomado algún medicamento para los parásitos intestinales?	○ No<br>○ Sí<br>○ NS/NR	

Explique a la madre que ahora, va a hacer algunas preguntas acerca de las vitaminas durante el embarazo.

Vitaminas durante el embarazo

24.	Durante su embarazo ¿le han dado Sulfato Ferroso?	○ No<br>○ Si<br>○ NS/NR	En caso de SI llenar también la 26
25.	Durante su embarazo, ¿tomó Ud. Sulfato Ferroso?	○ No<br>○ Sí<br>○ NS/NR	En caso de no→32
26.	¿De dónde obtuvo el Sulfato Ferroso?<br><br>No lea las opciones listadas en voz alta.<br><br>**Marque todas que aplican.**	☐ De clínica o centro de salud<br>☐ De farmacia<br>☐ De vecino/amigo/familia<br>☐ Compré en el mercado/tienda/calle<br>☐ Otro________________<br>☐ NS/NR	
27.	¿Con qué frecuencia tomaba el Sulfato Ferroso?<br><br>Selecciona solamente una unidad de tiempo que corresponde con el número	_______(#) veces<br>○ Diariamente (todos los días)<br>○ Semanal (veces a la semana)<br>○ Mensual (veces al mes)	

		○ Menos frecuentemente<br>○ NS/NR	
28.	En general, ¿fue fácil tomar las pastillas de Sulfato Ferroso?	○ No<br>○ Sí<br>○ NS/NR	
29.	¿Piensa que fue apropiada la frecuencia de tomar el Sulfato Ferroso?	○ No<br>○ Sí<br>○ NS/NR	En caso de sí→33
30.	¿Con qué frecuencia preferiría tomar el Sulfato Ferroso?<br><br>No lea las opciones listadas en voz alta.	○ Diariamente<br>○ Semanal<br>○ Mensual<br>○ Otro ______________<br>○ NS/NR	
31.	¿Qué sugeriría cambiar sobre la pastilla de Sulfato Ferroso?<br><br>No lea las opciones listadas en voz alta.<br><br>Marque todas que aplican.	○ Nada<br>○ El tamaño<br>○ El sabor<br>○ El olor<br>○ Manera de tomarlo (masticar vs. tragar)<br>○ Otro ______________<br>○ NS/NR	Cualquier respuesta →33

32.	¿Por qué no lo ha tomado?<br><br>No lea las opciones listadas en voz alta.<br><br>Marque todas que aplican.	○ No me dieron en la clínica<br>○ Me olvidé<br>○ Fue difícil tomar<br>○ Tenía mal sabor<br>○ Me causó dolor de estómago<br>○ Me causó estreñimiento<br>○ No recibí instrucciones<br>○ No sabía que era gratis<br>○ No tuve confianza en el doctor<br>○ No sabía para qué era<br>○ No sabía si habría riesgos<br>○ Prefiero tomar mis vitaminas desde la comida y no de pastillas<br>○ Otro_________________<br>○ NS/NR	
33.	Durante su embarazo, ¿tomó Ud. otros suplementos vitamínicos?	○ No<br>○ Sí<br>○ NS/NR	En caso de no→35

34.	En caso de sí, ¿cuáles fueron las otras vitaminas?	________________________<br>________________________<br>9999 La madre no sabe	

Vitaminas después del parto

35.	**[Primero del carnet]** Después del parto, ¿le han dado Vitamina A en el centro / clínica / hospital?<br><br>Anote la fuente de la información. Muestre la imagen de la pastilla, si es necesario.	○ No<br>○ Sí<br>○ NS/NR<br>Fuente:<br>○ Del carnet<br>○ De la madre	
36.	Después de su parto, ¿le han dado Sulfato Ferroso?<br>Muestre la imagen otra vez, si es necesario	○ No<br>○ Sí<br>○ NS/NR	En caso de SI llenar también la 38
37.	Después de su parto, ¿tomó Sulfato Ferroso?	○ No<br>○ Sí<br>○ NS/NR	En caso de no→41
38.	¿De dónde obtuvo el Sulfato Ferroso?<br><br>**No lea las opciones listadas en voz alta.**<br><br>**Marque todas que aplican.**	○ De clínica o centro de salud<br>○ De farmacia<br>○ De vecino/amigo/familia<br>○ Compré en el mercado/tienda/calle<br>○ Otro<br>○ NS/NR	

39.	¿Con qué frecuencia toma/ tomaba el Sulfato Ferroso?<br><br>Selecciona solamente una unidad de tiempo que corresponde con el número	_______(#) veces<br>○ Diariamente (todos los días)<br>○ Semanal (veces a la semana)<br>○ Mensual (veces al mes)<br>○ Menos frecuentemente<br>○ NS/NR	
40.	En los últimos 7 días, ¿Cuántas pastillas de Sulfato Ferroso ha tomado?	_________ (número de pastillas)<br><br>9999 La madre no sabe	Respuesta de 1 y mas→42
41.	¿Por qué no lo ha tomado el Sulfato Ferroso?<br><br>No lea las opciones en voz alta. Marque todas que aplican.	○ No me dieron en la clínica<br>○ Me olvidé<br>○ Fue difícil tomar<br>○ Tenía mal sabor<br>○ Me causó dolor de estómago<br>○ Me causó estreñimiento<br>○ No recibí instrucciones<br>○ No sabía que era gratis	

		○ No tuve confianza en el doctor<br>○ No sabía para qué era<br>○ No sabía si habría riesgos<br>○ Prefiero tomar mis vitaminas desde la comida y no de pastillas<br>○ Otro_________________<br>○ NS/NR	
42.	Después de su parto, ¿tomó otros suplementos vitamínicos?	○ No<br>○ Sí<br>○ NS/NR	Si no→fin
43.	¿Cuáles fueron las otras vitaminas?	___________________________________<br>___________________________________<br>9999 La madre no sabe	

VISITA 1

ID del estudio: _________

Encuesta de Morbilidad Materna

Ahora, quisiera hacerle unas preguntas acerca de su salud en las últimas dos semanas.

Nro.	Pregunta	Respuesta	Patrón de saltos
1.	En las últimas dos semanas, ¿ha tenido diarrea?	○No<br>○ Sí<br>○NS/NR	
2.	En las últimas dos semanas, ¿ha tenido tos o problemas respiratorios?	○ No<br>○ Sí<br>○NS/NR	
3.	En las últimas dos semanas, ¿ha tenido fiebre?	○ No<br>○ Sí<br>○NS/NR	
4.	En las últimas dos semanas, ¿ha estado Ud. hospitalizada?	○ No<br>○ Sí<br>○NS/NR	En caso de no→7
5.	¿Por cuantos días estuvo hospitalizada?	_______ días<br>9999 La madre no sabe	
6.	¿Por qué estuvo usted hospitalizada?	○ Problemas respiratorios<br>○ Diarrea<br>○ Otro ____________________________<br>○NS/NR	
7.	En las últimas dos semanas, ¿ha tenido algunos otros problemas de salud?	○ No<br>○ Sí<br>○ NS/NR	En caso de no→fin
8.	¿Cuáles son los otros problemas de salud que ha tenido?	1.<br>2.<br>3.	

VISITA 1 ID del estudio: _________

Encuesta: Seguimiento nutricional del niño y Madre

Ojo: El texto en gris es una nota para que el encuestador tenga en cuenta

(Suplemento Visita 1)

Nro.	*Pregunta*	*Respuesta con Códigos*	*Patrón de saltos*
1.	¿ Le ha dado pecho a su bebé?	○ No<br>○ Sí	En caso de no→fin de sección pase a la 3
2.	¿Cuándo se le dio al bebé pecho por primera vez después del nacimiento?<br>**No lea las opciones listadas en voz alta.**<br>Marque la que corresponde con la respuesta de la madre.	○ < 1 hora<br>○ 1 hora -3 horas<br>○ >3 horas<br>○ NS/NR	

(Visitas 1-8)

3.	¿En las últimas 24 horas, le ha dado pecho a su bebé?	○ No<br>○ Sí	
4.	¿Hasta el día de hoy, le ha dado al bebé algún líquido además de la leche materna?	○ No<br>○ Sí	Si no→ 7
5.	¿Qué liquido le ha dado al bebé además de la leche materna?<br>**<u>NO</u> lea las opciones listadas en voz alta.**<br>Marque todas que apliquen.	○ Leche de fórmula<br>○ Leche de vaca<br>○ Agua<br>○ Té/Mate<br>○ Jugo<br>○ Gaseosa<br>○ Otro _______________	
6.	¿Cuántas veces le dio líquido(s), que no sea leche materna, al bebé en las últimas 24 horas?	_______(#) veces<br>9999 La madre no sabe	
7.	¿Hasta el día de hoy, le ha dado al bebé alguna alimentación complementaria además de la leche materna?	○ No<br>○ Sí<br>○ NS/NR	En caso de no→ 10
8.	¿Qué alimentación complementaria le ha dado al bebé además de la leche materna?<br>No lea las opciones listadas en voz alta.<br>Marque todas que apliquen.	○ Yogurt<br>○ Papilla<br>○ Otro _______________<br>○ NS/NR	
9.	¿Cuántas veces le dio esta(s) alimentación(es) complementaria(s) al bebé en las últimas 24 horas?	_______(#) veces<br>9999 La madre no sabe	
10.	¿Ha recibido u obtenido jarabe o gotas de hierro para su bebé?<br>Mostrar imagen	○ No<br>○ Sí<br>○ NS/NR	En caso de no o no se→fin de

			la sección
11.	¿Dónde recibió u obtuvo este hierro para su bebé?	○ De clínica o centro de salud<br>○ De farmacia<br>○ De vecino/amigo/familia<br>○ Compré en el mercado/tienda/calle<br>○ Otro<br>○ NS/NR	
12.	¿Cuántas veces en la última semana tomó su hijo este jarabe o gotas de hierro?	_______(#) veces al semana<br>9999 La madre no sabe	Ante respuesta de 1 o más FIN
13.	En caso de cero (0) veces, ¿Por qué no ha tomado el hierro?<br>**No lea las opciones listadas en voz alta.**<br>Marque todas que apliquen.	○ Me olvidé darle<br>○ No recibí instrucciones<br>○ No sabía que era gratis<br>○ No sabía para qué era<br>○ No sabía si habría riesgos<br>○ El bebé estaba llorando y no quiso tomarlo<br>○ El bebé gesticuló que no quería<br>○ El bebé lo vomitó/escupió<br>○ Otro_________________<br>○ NS/NR	

Aceptabilidad de Chispitas (Visita 1)

Nro.	*Pregunta*	*Respuesta con Códigos*	*Patrón de saltos*
1.	¿Ha oído hablar de las Chispitas?<br>Muestre la bolsita	○ No<br>○ Sí<br>○ NS/NR	En caso de no→fin de sección
2.	¿En su conocimiento, para qué se utilizan Chispitas?<br>**No lea las opciones listadas en voz alta.**<br>Marque todas que apliquen.	○ Prevenir la anemia<br>○ Para que el bebé este más sano<br>○ Darle energía al bebe<br>○ Otro __________<br>○ NS/NR	
3.	Si tiene otros hijos, ¿Le(s) dio Chispitas a sus otros hijos cuando eran pequeños?	○ No<br>○ Sí<br>○ No tiene otros hijos<br>○ NS/NR	En caso de no o no tiene otros hijos →fin de sección
4.	En general, ¿Cómo fue su experiencia con las Chispitas?<br>**No lea las opciones listadas en voz alta**	○ No le gustó<br>○ Neutral<br>○ Si le gustó	

		○ NS/NR	
5.	En su opinión, ¿Cuáles son algunas características que no le gustaron sobre el uso de Chispitas?<br><br>**No lea las opciones listadas en voz alta.** Marque todas que apliquen.	○ Ninguna, no hubo nada que no me gustara<br>○ El sabor de la comida con chispitas<br>○ El olor de la comida con chispitas<br>○ La consistencia de la comida con chispitas<br>○ El color de la comida con chispitas<br>○ El tamaño del paquete<br>○ La cantidad en cada paquete<br>○ Era difícil usar<br>○ Tomó tiempo preparar con la comida<br>○ Le causó malestar al bebe<br>○ Le causo diarrea al bebe<br>○ Causo estreñimiento<br>○ Se olvidó darles<br>○ Otro ______________<br>○ NS/NR	

6.	En su opinión, ¿Cuáles son algunas características que le gustaron sobre el uso	○ Ninguna, pues nada me gusto	

	de Chispitas?<br><br>No lea las opciones listadas en voz alta. Marque todas que apliquen.	○ El sabor de la comida con Chispitas<br>○ El olor de la comida con Chispitas<br>○ La consistencia de la comida con Chispitas<br>○ El color de la comida con Chispitas<br>○ El tamaño del paquete<br>○ La cantidad en cada paquete<br>○ Era fácil usar<br>○ No tomó mucho tiempo preparar con la comida<br>○ Otro ______________<br>○ NS/NR	
7.	¿Usted ha probado las Chispitas?	○ No<br>○ Sí	
8.	Dado sus respuestas sobre las características buenas y malas de las chispitas, ¿cree que las mujeres embarazadas **podrían** usar un producto como Chispitas regularmente?	○ No<br>○ Sí<br>○ NS/NR	
9.	Dado sus respuestas sobre las características buenas y malas de las chispitas, ¿cree que las mujeres embarazadas **quisieran** usar un producto como Chispitas regularmente?	○ No<br>○ Sí<br>○ NS/NR	

VISITA 1

ID del estudio: _________

Encuesta de Morbilidad y Diarrea Infantil

Visitas 1-8

Ahora, quisiera hacerle unas preguntas acerca de su salud de su bebé en las últimas dos semanas.

1.	En las últimas dos semanas, ¿ha tenido tos o problemas respiratorios?	○ No<br>○ Sí<br>○ NS/NR	
2.	En las últimas dos semanas, ¿ha tenido fiebre?	○ No<br>○ Sí<br>○ NS/NR	
3.	En las últimas dos semanas, ¿ha estado Hospitalizado su bebé?	○ No<br>○ Sí<br>○ NS/NR	En caso de no o NS/NR →6
4.	¿Por cuantos días estuvo hospitalizado?	_______ días<br>9999 La madre no sabe	
5.	¿Por qué estuvo el niño(a) hospitalizado?	○ Problemas respiratorios<br>○ Diarrea<br>○ Otro ____________________<br>○ NS/NR	
6.	En las últimas dos semanas, ¿ha tenido algunos otros problemas de salud?	○ No<br>○ Sí<br>○ NS/NR	En caso de no→8
7.	¿Cuáles son los otros problemas de salud que ha tenido?	1.<br>2.<br>3.	

Recordatorio de Diarrea

La diarrea infantil se define como un número mayor y anormal de deposiciones para un bebeen un periodo de 24 horas. (**Verificar si la mamá sabe lo que es diarrea)**

Nro.	*Pregunta*	*Respuesta*	*Saltos*
8.	¿Cuándo está saludable el bebe, cuántas deposiciones diarias es normal para el/ella? Si no recuerda o no responde, ponga "99"	____ ____ deposiciones	
9.	En las últimas dos semanas, ¿ha tenido diarrea su bebé?	○ No<br>○ Sí<br>○ No sabe/no responde	En caso de no o no sabe o no responde → 18

10.	¿Hace cuántos días empezó la diarrea?	____ ____ días	
11.	¿Hace cuántos días terminó la diarrea, o todavía sigue? **Si todavía sigue, escribe "99"**	____ ____ días	
12.	¿En el peor día de su diarrea, cuántas deposiciones tuvo el bebé? Si no recuerda o no responde, ponga "99"	____ ____ veces	
13.	¿Cuándo tuvo diarrea esta vez, hubo sangre en su caquita?	○ No<br>○ Sí<br>○ No sabe/no responde	
14.	¿Cuándo tuvo diarrea esta vez, hubo vómitos?	○ No<br>○ Sí<br>○ No sabe/no responde	En caso de no salto a la 16
15.	¿En el peor día de su diarrea cuantas veces vomito?	_____ _____ veces	
16.	¿Hizo algo usted para que el bebe mejore?	○ No<br>○ Sí<br>○ No sabe/no responde	En caso de no o no sabe o no responde →18
17.	¿Qué hizo?<br>**No lea las opciones listadas. Marque todas que apliquen**	□ Llevarlo a la clínica<br>□Líquidos caseros (agua de arroz, canela, etc.)<br>□Solución casera (agua con sal y azúcar)<br>□ Sales de rehidratación oral<br>□Medicamento contra la diarrea (Ej. jarabe de zinc)<br>□ Antibiótico/Antiparasitario<br>□ Remedio de hierbas<br>□Otro: ___________	
18.	¿Le ha dado un nuevo alimento o liquido al bebe, o ha cambiado la dieta en las últimas dos semanas?	○ No<br>○ Sí<br>○ NS/NR	
19.	¿Alguien más en su casa ha tenido diarrea en las últimas dos semanas?	○ No<br>○ Sí<br>○ No sabe/no responde	**En caso de no FIN**

20.	¿Quién(es) ha(n) tenido diarrea?<br>**No lea las opciones listadas. Marque todas que apliquen**	□Otro niño/a en la vivienda<br>□Madre del niño<br>□Padre del niño<br>□ Abuelo/a del niño<br>□ Otro adulto en la casa<br>□ Otro:____________	

Si su hijo desarrolla diarrea, por favor llame a la responsable del hospital para poder recolectar el pañal con caquita.

VISITA 1 ID del estudio: _________

MADRE: Toma de muestra de sangre

Ojo: El texto en gris es una nota para el encuestador tener en cuenta

[ ]

Etiqueta de muestra de sangre

Verifique que el número en esta etiqueta es el mismo del estudio ID del paciente

Nro.	*Pregunta*	*Respuesta con Códigos*	*Patrón de salteos*
1.	Hora de la toma de muestra	__ __: __ __<br>h h : m m<br>99:99 No sabe	
2.	Hemoglobina (g/dL) (nivel bruto/observado)	☐☐ . ☐g/dL<br>*Marque el resultado de Hb también en la tarjeta de resultados NIDI de la madre*<br>99.9Medida no tomada	
3.	Se obtuvo muestra para:	○ Hemoglobina<br>○ Análisis<br>○ Las dos anteriores<br>○ No se obtuvo muestra	
4.	¿Hubo alguna injerencia con las medidas?	○ No<br>○ Sí, observaciones: ____ _________________	
5.	¿Actualmente está embarazada?	○ No<br>○ Sí<br>○ NS/NR	
6.	¿Ha tenido fiebre en las últimas 48 horas?	○ No<br>○ Sí<br>○ NS/NR	
7.	¿A qué hora comio su último alimento?	__ __: __ __<br>h h : m m<br>99:99 La madre no sabe<br>○ hoy<br>○ ayer<br>○ NS/NR	

VISITA 1

ID del estudio: _________

Con el resultado obtenido determinar la presencia de anemia según la Tabla 1 debajo.

Población	Anemia
Mujeres NO embarazadas	Hb menos de 14.7 dg/L
Mujeres embarazadas	Hb menos de 13.7 dg/L

8.	¿Tiene anemia la madre?	○ No<br>○ Sí Márquelo en:<br>1) el registro de referencia de anemia<br>2) boleta de referencia<br>3) Registro de laboratorio<br>4) Hoja de evolución	

Camine con la paciente hasta la sala de espera. Pregunte si se siente bien. ¿El paciente presenta alguno de los siguientes síntomas?

	Sí	No
Moretón en el sitio de extracción de la sangre	○	○
Inflamación en el sitio de extracción de la sangre	○	○
Sangrado excesivo en el sitio de extracción de la sangre	○	○
Desmayo	○	○
Muerte (informe a la Dra. Rita Revollo inmediatamente)	○	○
Otro problema posiblemente relacionado con la toma de sangre:	○	○

____

Si alguna de las complicaciones mencionados ocurrieron a una madre o al niño(a) después de la extracción de sangre(excepto moretón):

1. **Refiérase la madre a un médico para atención con el boleto de referencia.**

2. **Anote el evento adverso usando la encuesta y registro de Eventos Adversos.**

3. **Si ocurre una muerte, informe a la Dra. Rita Revollo inmediatamente e inicie protocolo de Eventos Adversos.**

Diga a la mama del participante que puede irse. Instrúyale que si tiene complicaciones o problemas durante los próximos días, que llame a la coordinadora del proyecto, cuya nombre y número está en el carnet del estudio ID les dimos.
